# Reliable Control Applications with Wireless Communication Technologies: Application to Robotic Systems

**DOI:** 10.3390/s21217107

**Published:** 2021-10-26

**Authors:** Isidro Calvo, Eneko Villar, Cristian Napole, Aitor Fernández, Oscar Barambones, José Miguel Gil-García

**Affiliations:** 1 System Engineering and Automation Deparment, Faculty of Engineering of Vitoria-Gasteiz, Basque Country University (UPV/EHU), 01006 Vitoria-Gasteiz, Spain; evillar005@ikasle.ehu.eus (E.V.); cristianmario.napole@ehu.eus (C.N.); afernandez458@ikasle.ehu.eus (A.F.); 2 Department of Electronic Technology, Faculty of Engineering of Vitoria-Gasteiz, Basque Country University (UPV/EHU), 01006 Vitoria-Gasteiz, Spain; jm.gil-garcia@ehu.eus

**Keywords:** industrial internet of things (IIoT), wireless control systems, industry 4.0, XBee, LabVIEW, fuzzy control, reliable control systems, robotics, gain scheduling fuzzy PID controller

## Abstract

The nature of wireless propagation may reduce the QoS of the applications, such that some packages can be delayed or lost. For this reason, the design of wireless control applications must be faced in a holistic way to avoid degrading the performance of the control algorithms. This paper is aimed at improving the reliability of wireless control applications in the event of communication degradation or temporary loss at the wireless links. Two controller levels are used: sophisticated algorithms providing better performance are executed in a central node, whereas local independent controllers, implemented as back-up controllers, are executed next to the process in case of QoS degradation. This work presents a reliable strategy for switching between central and local controllers avoiding that plants may become uncontrolled. For validation purposes, the presented approach was used to control a planar robot. A Fuzzy Logic control algorithm was implemented as a main controller at a high performance computing platform. A back-up controller was implemented on an edge device. This approach avoids the robot becoming uncontrolled in case of communication failure. Although a planar robot was chosen in this work, the presented approach may be extended to other processes. XBee 900 MHz communication technology was selected for control tasks, leaving the 2.4 GHz band for integration with cloud services. Several experiments are presented to analyze the behavior of the control application under different circumstances. The results proved that our approach allows the use of wireless communications, even in critical control applications.

## 1. Introduction

Modern Industry 4.0 applications improve the productivity, efficiency, safety and intelligence of the production processes. Typically, they involve different operations such as monitoring, predictive maintenance, supervision and control operations [1,2]. In this scenario, Cyber Physical Production Systems (CPS/CPPS) [3] play a key role. Communication capabilities are one of the key dimensions identified at the development of CPS/CPPS [4,5] since they must connect the cyber platforms with the physical world providing adequate Quality of Service (QoS) parameters. In particular, wireless technologies are gaining increasing relevance in production processes [6,7,8], since they allow deployment flexibility, support to mobile devices, reducing cabling and improving scalability. Industrial applications require bounding certain QoS parameters, in particular delay, jitter and error rate. Unfortunately, the nature of wireless wave propagation may reduce the reliability and integrity of the communication links when compared to wired links. Industrial facilities, full of metallic surfaces and obstacles, may suffer especially diverse phenomena such as signal fading, interferences and propagation by different paths. However, although factory automation tends to be quite conservative, the expected benefits of introducing wireless technologies are considered to outgrow the inconveniences. But the use of wireless technologies in industrial applications is still under scrutiny, especially at critical use cases [9]. They are starting to be introduced for some operations depending on the required values for the latency and jitter [10]. Typically, they are accepted in monitoring, condition alarming and supervisory control operations, with relatively low sampling frequencies, but they are more problematic for critical operations such as feedback control or safety operations [11].

Conversely, the influence of interferences caused by uncoordinated communication technologies, needs to be minimized. Most low power wireless technologies used at industrial applications, e.g., ZigBee, Bluetooth, ISA 100.11a WirelessHART and WIA-PA, operate at the 2.4 GHz frequency band [12,13]. The introduction of some techniques may improve coexistence among these technologies, but they are still vulnerable to transmissions from uncoordinated stations in the 2.4 GHz band. Wi-Fi 2.4 GHz technology is particularly problematic since it is commonly found in industrial facilities and uses a transmission power 100 times higher than typical IEEE 802.15.4 nodes [14]. QoS degradation at industrial applications due to Wi-Fi interferences with other technologies was checked in [15,16,17]. Zigbee is one of the few mainstream low power wireless technologies that can use frequency bands different from 2.4 GHz [18]. This technology was selected in this work to provide an independent communication channel without interferences with Wi-Fi. Namely, XBee 900 MHz modules, based on the IEEE 802.15.4 protocol, were chosen.

This work focuses on using wireless communications for closed-loop regulatory control operations. Control operations require periodic feedback for smooth running of the processes in which sensors and actuators are involved. According to [7], these operations produce a significant density in the network traffic for two reasons: (1) high sampling rates are used which generate periodic traffic and (2) control systems try to minimize the dead-time between two consecutive communications to optimize the performance of the control algorithm. Since failures in communication may lead to the instability of the process control, high reliability must be achieved. Unfortunately, wireless links are less predictable than wired links. As a consequence, wireless control systems must be designed in a holistic way considering both the communications QoS and the control algorithm since they are intertwined [19,20,21]. For example, the sampling period must be adequately chosen such that it does not disturb the control algorithm. In addition, some specific policies must be introduced to cope with message delays, jitter, message dropouts or total loss of the wireless communication link, since they may appear occasionally when using wireless communication technologies.

**Figure 1:**
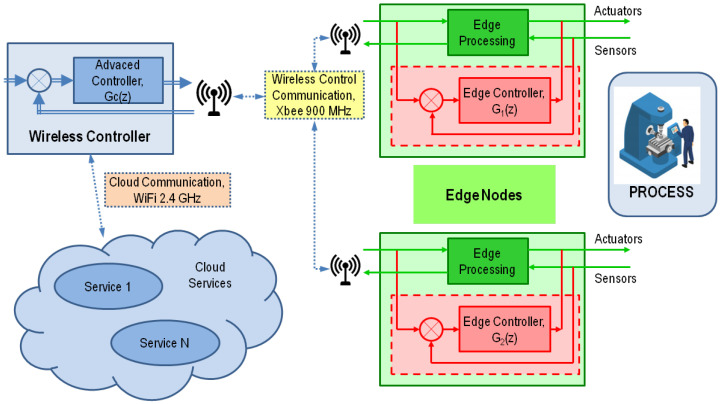
Overall wireless architecture for reliable edge computing.

More specifically, this work presents a wireless control scheme aimed at achieving reliable wireless control systems even in the case of communication degradation or total loss of the communication link. The presented control approach is based on the use of two levels of controllers: sophisticated control algorithms, providing better performance, are executed in a central node, while back-up controllers, implemented as local independent controllers, are executed next to the process in case of QoS degradation in the communication links. An operating architecture with two types of nodes was defined (see Figure 1): the so-called Wireless Controller and Edge Nodes. The Wireless Controller, implemented in a central node, is able to execute advanced control algorithms, requiring higher computing resources. Edge Nodes, located next to the process, will take over in case of QoS degradation by executing back controllers implemented over low computing resources platforms. The presented approach presents a reliable strategy for switching between central and local controllers avoiding that the system becomes uncontrolled and recovering the central controller when the communication QoS parameters are considered safe. For validation purpose, the control scheme was implemented over a NI myRIO 1900 as Wireless Controller and an ad hoc board powered with an Arduino MKR Zero as Edge Node. The design of the application protocol used by these devices is also discussed.

An independent communication channel was used for control communication operations. Namely, XBee 900 MHz modules were used. This technology minimizes the interferences caused by other technologies, typically operating at the 2.4 GHz band [12,13], and leaves the 2.4 GHz band available for connecting to cloud services with less-critical requirements. In this work, XBee 900 MHz modules were validated for control applications by means of several experimental tests.

The presented approach was validated by means of a planar robot, emulated as Hardware in the Loop plant and connected to the Edge Node by means of the SPI bus. An advanced control algorithm, based on Fuzzy Logic, was executed at the Wireless Controller, whereas a low resource PID was executed as backup controller at the Edge Node. Experimental results proved that the planar robot did not become uncontrolled, even in case of QoS degradation or even total communication loss. These results may be extended to other plants, such that the current case study may serve as a guide to introduce wireless technologies to critical control applications.

The layout of the article is as follows. Section 2 discusses related works. Section 3 presents the architecture for the wireless control scheme and validates experimentally XBee 900 MHz technology. Section 4 presents the followed approach for implementing reliable wireless control systems. Section 5 illustrates, by means of a case study, based on a planar robot, the procedure to implement the presented approach. Finally, the article draws conclusions.

## 2. Related Work

The IEEE 802.11 standard is not adequate for stringent, deterministic industrial applications but sometimes is used. Experimental amendments were proposed in [22,23]. The incipient IEEE 802.11ah standard, defined in 2017, is aimed to support IoT applications [24]. This standard operates in the 900 MHz band, and it is being analyzed for control tasks [25]. Although several prototypes have been developed, it still is rare to be deployed at industry facilities.

However, the majority of industrial standards, i.e., ZigBee, WirelessHART, ISA100.11a and WIA-PA [17], are based on IEEE 802.15.4. The performance of this standard at sensor networks for industrial applications has been analyzed in [26,27]. The incipient IEEE 802.15.4e standard is aimed at industrial, commercial, and healthcare applications [28], but still, it needs to gain more acceptation. Several amendments aimed at improving reliability and latency predictability were presented in [29,30]. WirelessHART, based over IEEE 802.15.4, is a promising technology which is gaining acceptation at industrial applications since it introduces higher reliability due to the use of the TSCH CSMA-CA algorithm [29,31,32]. New technologies, such as 5G, look promising, since they ensure low latencies, around 1 ms, but they are often discarded since currently they are still expensive [33]. They may constitute an interesting alternative to cover manufacturing plants when combined with other technologies [34].

In this scenario, ZigBee is one of the most popular technologies based on the IEEE 802.15.4 standard. This technology has been used in different applications at different domains. In the power electricity domain, it has been used for sending messages from intelligent electronic meters to concessionaires [35], in combination with CAN technology to control a photovoltaic based AC Microgrid [36], or for energy management, efficiency optimization and metering services within a smart grid [37]. It was also employed indoors and outdoors for controlling robots or machinery in the range of several meters. For example, for monitoring and controlling robotics hand remotely or using hand gestures [38,39]. In addition, for implementing a MPC algorithm aimed at a multi-drone system [40] or, for controlling an in-wheel motor by means of a network-based discrete proportional integral (PI) algorithm [41]. In [15], it was used to control remotely a piezoelectric actuator. Some examples are aimed at Industrial IoT applications: a monitoring architecture for process automation technologies is presented in [18], and a wireless sensor network for an assembling line [42]. One of the advantages of the ZigBee technology is the availability of mature and low cost products, such as the XBee series, that may operate even at the 868 and 900 MHz frequency bands. The use of technologies in these bands may provide some advantages, such as operating at longer distances, and should be analyzed carefully. Applications that employ these modules may be found in [43,44,45].

The main challenge in wireless control system is to jointly design the communication and control systems considering their tight interaction to improve the control performance and the network lifetime, since controllers should ensure the required dynamic and steady state response [46]. Critical variables identified in [19] were: sampling period, message delay, message dropout and energy consumption; thus, these variables must be jointly tuned. Three control approaches were discussed in this article, namely Proportional-Integral-Derivative (PID), Linear Quadratic Regulator (LQR) and Model Predictive Control (MPC) [47]. Some authors propose the use of estimators [48] and filtering techniques [49] to compensate communication delays and dropouts. Thus, the current status of the plant may be predicted based on known states.

New paradigms, such as cloud computing, are being introduced in Industry 4.0 to introduce different types of services by means of computer networks, typically based on TCP/IP technologies [50]. This paradigm introduces higher flexibility at the applications allowing sophisticated off-line analysis of process data. However, the use of non-deterministic communication technologies limits the possibilities of this paradigm. In this scenario, other approaches were proposed. For example, the Edge Computing paradigm allows connecting a great number of IoT devices to the cloud [51]. In edge computing, the processing is shifted to the edge of the network, such that application requirements may be achieved, in terms of bounded time, battery life, saving bandwidth as well as improving security and privacy. Edge computing involves the devices connected to the sensors and actuators. Fog computing is a variation of Edge computing [52]. It distributes the execution of the algorithms, communication, control and data logging in infrastructures owned by the companies. In this scenario, it is key to provide connectivity between the industrial processes and cloud services. Several works in the literature propose different architectures that allow the integration of process data acquired with ZigBee [16,53,54].

## 3. Wireless Control System for Edge Systems

This section focuses on the architectural design of wireless control systems with cloud connectivity. It discusses the requirements of the addressed control systems. In addition, a control architecture, based on two different types of nodes, the so-called Wireless Controller and Edge Nodes is proposed. This section also discusses the implementation with selected technologies. Finally, XBee technology is analyzed in order to know the QoS requirements of the proposed approach.

Our approach is aimed at control systems that operate at sampling periods in the range from 25 to 100 ms, with bounded delays. Expected distances between the Edge nodes, attached to the process, and the Wireless Controller, which executes the control algorithm, are in the range of a few meters. This distance is considered adequate for a large number of industrial applications, involving robotic applications since it has been used in several works [18,38,39,41,42,45]. For instance, Dalef et al. [38] provided experimental results of wireless control and monitoring design for a remote located robotic hand. Authors of [39] used wireless control for quadcopters where the distance is important factor in the design. Another study was conducted by Xie et al. [45], for remote locations of energy plants by wind features sensing. In addition, our approach must be compatible with the operation of 2.4 GHz Wi-Fi technology since this a popular alternative for connecting industrial processes with cloud services in the Industry 4.0 paradigm. Finally, different types of analog and digital sensors and actuators must be allowed.

### 3.1. Wireless Architecture for Reliable Edge Computing

The presented wireless control approach is based on two types of differentiated nodes, the *Wireless Controller*, which executes remotely an advanced control algorithm of the process, and the *Edge Nodes*, which are directly connected to the processes by means of several sensors and actuators (see Figure 1).

#### 3.1.1. Wireless Controller

A Wireless Controller is responsible for executing the control algorithm of the plant. It must be implemented over an adequate platform for control, such as a real-time embedded platform, with a precise real-time clock. The platform must provide adequate computing power and programming capabilities for implementing sophisticated control algorithms based on advanced control techniques such as Artificial Neural Networks, Fuzzy Logic or Model Predictive Control (MPC). Since our approach is addressed to industrial applications, platforms accepted in industrial scenarios should be used. In some cases, it may be convenient to combine both wireless and wired links; thus, the chosen platform should be able to allow different types of connections with process devices. The Wireless Controller may be used as intermediate device for connecting industrial processes to cloud services, typically using a different technology such as Wi-Fi 2.4 GHz.

#### 3.1.2. Edge Nodes

They are directly connected to the process by means of several sensors and actuators. They perform the following operations: (1) acquire the values of the sensors and preprocess them, if necessary; (2) provide communication capabilities with the Wireless Controller; (3) receive the control signal to set the actuators; and (4) detect communication problems and react accordingly to avoid that the process reaches an undesired state. Edge Nodes require using a low resource computing platform. In addition, Edge Nodes must be configured remotely to ease its deployment. Edge nodes are expected to ease the connection to industrial sensors and actuators. Finally, they should be able to be powered with batteries to allow being located in difficult to reach places or moving parts.

#### 3.1.3. Wireless Connectivity

Wireless communication must be able to provide the QoS requirements of the application, which depend on the dynamics of the processes to control. In our approach, we are targeting systems which are expected to operate with sampling times of around 25 to 100 ms and jitter of around 1 ms. In addition, in the Industry 4.0 paradigm, the Wireless Controller typically needs to be connected to the cloud, may be a private cloud, for additional services that may involve vertical and horizontal integration or process logging. This fact recommends the use of a different communication band to the 2.4 GHz, frequently used by Wi-Fi in industrial facilities.

The majority of low power technologies currently used in industrial applications work at the 2.4 GHz band and when used for control operations they may suffer the interferences caused by Wi-Fi. The only low power-short range wireless technology available in the market which operates in a different band is ZigBee, which may operate at the 900 MHz band. For these reasons XBee 900 MHz technology, based on the IEEE 802.15.4 standard, was chosen for implementing the wireless control loop. It is a well-proven, robust and low-cost technology. In addition, it allows using Wi-Fi 2.4 GHz for vertical/horizontal integration and connection to cloud services.

### 3.2. Architecture Implementation

#### 3.2.1. Wireless Controller

A National Instruments (NI) myRIO 1900 device was used as Wireless controller. This is a real-time embedded board powered with a dual-core ARM Cortex-A9 and a Xilinx FPGA. It is a typical real-time embedded platform which features several analog and digital Inputs/Outputs. It runs on top of a Linux RTOS and it is programmed in LabVIEW. The NI myRIO provides similar features to the devices available at the CompactRIO series. These industrial devices allow the combination of a broad number of modules, including controllers, reconfigurable IO modules and communication technologies aimed at real-time embedded industrial systems. Several programming languages may be used, including LabVIEW, C, C++ or Java. The current approach could be implemented with robust devices typically used for industrial applications.

XBee modules are connected with a serial link, which can be easily managed from LabVIEW with the NI VISA device. LabVIEW also provides diverse blocks to implement different control schemes, including Fuzzy Logic and Artificial Neural Networks. The LabVIEW application is responsible for: (1) configuring the Edge Nodes at start time; (2) receiving the value of the remote sensors and check the integrity of the frames; (3) executing the control algorithm with the values of the sensors; and (4) sending the control signal to the Edge Nodes attached to the actuators.

#### 3.2.2. Edge Nodes

**Figure 2:**
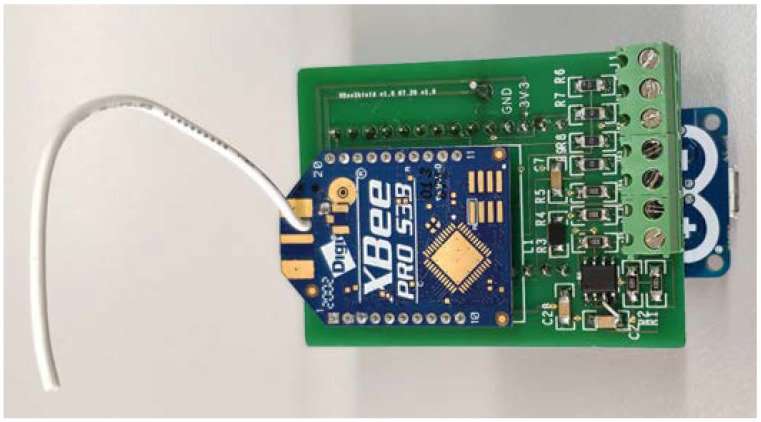
Detail of the Edge Node prototype board.

Edge Nodes were implemented with an ad hoc board which integrates commercial off-the-shelf components (See Figure 2). Namely, the edge processing was carried out by an Arduino MKR Zero board, based on a 32 bit SAMD microcontroller. It features seven analogue channels of twelve bits and supports a Li-Po single cell, 3.7 V battery along with an expansion connector compatible with the small form-factor Arduino pinout. XBee Pro S3B modules provide wireless communication to the Edge Node. An asynchronous (UART) port connects the Arduino MKR board with the XBee module. An ad-hoc printed circuit board (PCB) was specifically designed and manufactured for implementing the Edge Nodes. The board provides expansion connectors for easing the installation of up to four analogue sensors which should operate in the range 0–3.3 V. One of them can also be used as an analogue output. In addition, other sensors and actuators may be connected by means of the SPI bus, which is a short-distance, synchronous, serial communication interface specification for connecting digital sensors/actuators.

The microcontroller application does the following tasks: (1) configure the Edge Nodes at start time; (2) measure the values of the attached sensors on the reception of the measurement frames; (3) set the values of the actuators according to the control algorithm; (4) detect communication problems such as loss or wrong frames; and (5) take over the control of the process in case that the communication link is degraded or lost.

#### 3.2.3. XBee 900 MHz Communication

XBee, based on the IEEE 802.15.4 standard, was chosen to connect the Wireless Controller with the Edge Nodes. The XBee ecosystem is a set of compatible form factor wireless communication modules manufactured by Digi International. Several topologies may be defined depending on the requirements of the applications involving three types of devices: a unique Coordinator, Routers (if needed) and several End Devices. The Coordinator plays a fundamental role in the network as it manages all aspects of the communication and the network. End Devices are typically responsible for collecting data of sensors or driving actuators. Two programming modes may be used to define the communication frames depending on the complexity and requirements of the applications, the so-called AT mode and the Application Programming Interface (API) mode. The API mode, chosen in this work, provides higher flexibility to define the frames of the application protocol but requires programming at a deeper level. The size of the frames depends on the application protocol defined, being the maximum size for the frames of 256 bytes [55].

Although XBee is not typically used at critical applications, this technology is flexible and may be configured for different topologies and scenarios. The chosen configuration of the XBee modules fulfils the QoS requirements of the wireless control system.

XBee technology permits different topologies. Since the proposed architecture is centralized in the Wireless Controller, a star topology is a natural choice. In addition, this topology eases binding communication latency and jitter, which is a key issue at closed-loop control operations. If adequately managed, this topology may be used for controlling a plant, even when several Edge Nodes are involved. In the proposed configuration the XBee module at the Wireless Controller was set in the Indirect Message Coordinator mode. The XBee modules at the Edge Nodes were set as Indirect Message Pollers. Other parameters, such as the maximum number of hops across the network were set to 1. Unicast retries were disabled to achieve higher determinism. Broadcast multi-transmits and unicast retries were disabled. The transmission power of the XBee modules was set to the maximum value (+24dB). The serial communication for connecting XBee modules with the NI myRIO and Arduino MKR Zero respectively was set to a value of 115,200 bauds without parity, which was the higher operating value. This value achieved lower sampling periods. Table 1 summarizes most the relevant parameters used for the configuration of the XBee modules.

### 3.3. Experimental Validation of XBee 900 MHz for Control Applications

This section analyzes experimentally whether the XBee 900 MHz modules may achieve the necessary QoS requirements for the proposed wireless control scheme.

Several tests were carried out to characterize the QoS parameters of the XBee 900 MHz communication link between one NI myRIO device, used as Wireless Controller, and several Edge Nodes powered with Arduino MKR Zero boards. The main parameters that needed to be characterized to analyze the feasibility of the setup are the latency, deemed as the time it takes from the moment a packet is sent until it reaches its destiny, and the jitter, considered as the maximum variation of the latencies observed among the sent packages over a period. Namely, the following tests were carried out: (1) latency and jitter of XBee messages issued by the Wireless Controller; (2) number of lost/wrong XBee messages; (3) reception jitter of XBee broadcast frames at different Edge Nodes and; (4) latency and jitter of the XBee messages sent by the Edge Nodes using different frame lengths. The first three tests were repeated at different distances, ranging from less than 1 to 12 m, while the last test was carried out using different message sizes.

Experimental tests were carried out at the Faculty of Engineering premises, namely in a laboratory in which industrial equipment was available, such as robots and electric motors. In addition, metallic objects and computers were found. In this area an intensive use of the institutional Wi-Fi network was made by the students.

#### 3.3.1. Latency and Jitter of XBee Messages Issued by the Wireless Controller

The NI myRIO serialized periodically frames to the XBee module, which sent them to the XBee module located at the Edge Node. One oscilloscope was used to measure the values of latency and jitter. Time was taken since the NI myRIO serialized the message to its XBee module until the Arduino MKR Zero received it. The test lasted 10 min and frames were sent every 60 ms. The size of the frames was of 18 bytes. The oscilloscope persistent feature was used to capture the maximum and minimum values of the reception. This test was repeated at different distances.

Table 2 shows the values obtained. It can be appreciated that there is not a big difference for the distances used at the experiments, although in the longer distance tests there were objects in the middle and there was not eye of sight. Average delay was around 10 ms and jitter was around 1 ms. Only for longer distances, around 12 m, was the value of the jitter increased. These values may be valid at some control applications depending on the dynamics of the process.

#### 3.3.2. Number of Lost/Wrong XBee Messages

This test counted the number of lost or wrong messages sent between the NI myRIO and the Arduino MKR Zero. The error rate of the XBee communication link was experimentally measured. Messages were sent every 60 ms. Since the test lasted 20 min, a total number of 20,000 frames was sent. The test was repeated at different distances, even with a wall of brick in between the two nodes. The results are shown in Table 3. It can be appreciated that the number of lost messages is remarkably low, even with the brick wall that separated different rooms in the building.

#### 3.3.3. Reception Jitter of XBee Broadcast Frames at Different Edge Nodes

This test checked whether the XBee broadcast messages, sent by the NI myRIO, were received at the same time by different Edge Nodes powered with Arduino MKR Zero boards. This test was carried out with an oscilloscope to compare the reception time at three different Edge Nodes. The test lasted 5 min. It was repeated at different distances from 0 to 12 m. XBee broadcast messages were issued every 60 ms. Table 4 presents the obtained results for the maximum time difference at the reception of the three Edge Nodes. This value was below 1 ms for all tests. In this test, Edge Nodes were always placed relatively close among them.

#### 3.3.4. Latency and Jitter of the XBee Messages Sent by the Edge Nodes Using Different Frame Lengths

This test measures the values of the latency and jitter for the XBee messages sent by the Edge Nodes using different frame lengths. It can be appreciated that the transmission time remains always below 20 ms, even for relatively long frames (53 bytes). With regards to the jitter, the value was always below 1 ms. Tests were executed at a distance of 5 m. The results are shown in Table 5.

#### 3.3.5. Discussion

The experimental tests carried out with the XBee 900 MHz modules attached to the NI myRIO and Edge Nodes indicate that, the latency for the transmission of XBee messages depends on its length, but the transmission jitter is typically below 1 ms. Obtained results were according to the hardware specifications in the analyzed distances. Experimental tests prove that the transmission of one message takes between 10 and 20 ms each way, depending on the size of the message. Since control applications require at least two messages, one for the control signal and another for the sensors values, control applications with sampling times in the range of 25–40 ms could be implemented with this technology, in case that the process dynamics can tolerate jitter values of around 1 ms. These values may be valid for a broad range of control applications. In addition, XBee broadcast messages arrive at several nodes with a low time difference. Thus, the approach of using the transaction clock at the Wireless Controller for sending broadcast messages may be valid for implementing distributed control applications. Finally, the number of loss and wrong packages is low, especially when compared with the use of XBee 2.4 GHz technology [15]. This is partly due to the reduction of the interferences caused by other technologies that operate in the 2.4 GHz band. The approach of using XBee technology allows separating the traffic in different channels, one for control operations, with XBee 900 MHz and another for connecting to additional cloud services using Wi-Fi 2.4 GHz.

## 4. A Reliable Wireless Control Scheme

The nature of wireless technologies penalizes the performance of the communications due to diverse inherent phenomena, such as signal fading, interferences or propagation by different paths. These phenomena hinder the use of wireless communication in critical control systems, since a reduction in the communication QoS parameters may affect the performance of the control application.

This section discusses how to approach the design of wireless control systems. Particularly, it proposes a reliable wireless control scheme aimed at coping with the occasional loss of packages or even an occasional communication blackout in critical control applications. The proposed wireless control scheme defines which actions must be taken in both ends of the distributed system, i.e., the Wireless Controller as well as the Edge Nodes. These actions are aimed at avoiding that the wireless control system becomes uncontrolled. Several operating states, according to the current status of the communication links, are defined as well as the actions to be taken in every case. In addition, an application protocol was defined, which was implemented over XBee 900 MHz modules.

Our approach requires the use different controllers (see Figure 1). A more sophisticated, CPU demanding controller is executed at the Wireless Controller, in a powerful computing platform. The control algorithm calculates the control value according to the values of the process sensors and the reference for the system. Control values are sent by means of wireless links to the Edge Nodes. As shown in Section 3.3.3, the measured jitter in the reception of the broadcast messages at the different Edge Nodes remains below 1 ms. This value may be acceptable for lots of applications, since our approach is aimed at applications that use sampling periods in the range of several tens of ms. In case that the communication links are working properly, the calculated control value is used to set the control signal of the actuators. At the same time, the Edge Nodes acquire the attached sensor values and send them to the Wireless Controller to execute the control algorithm.

In case that wireless links do not operate properly, i.e., messages are lost or even a communication blackout comes up, the Edge Nodes take over the control tasks. For that purpose, a low computational cost controller must be implemented at the Edge Nodes in order to perform control tasks, obviously with a lower performance.

One important issue is detecting when the link is considered lost. Sometimes, the loss of just a few frames does not affect the performance of the plant, especially if the wireless control algorithm is robust enough to recover the expected behavior for the process. However, typically frames are lost in bursts. In such case, the Edge control algorithm must be executed, taking over the Wireless Controller. When the wireless communication is recovered and some conditions are satisfied, the control scheme executes back the wireless control algorithm.

Finally, our wireless control approach uses a centralized mechanism for configuring the distributed Edge Nodes. The configuration of the nodes is issued from the Wireless Controller.

### 4.1. Mode Change State Diagram

**Figure 3:**
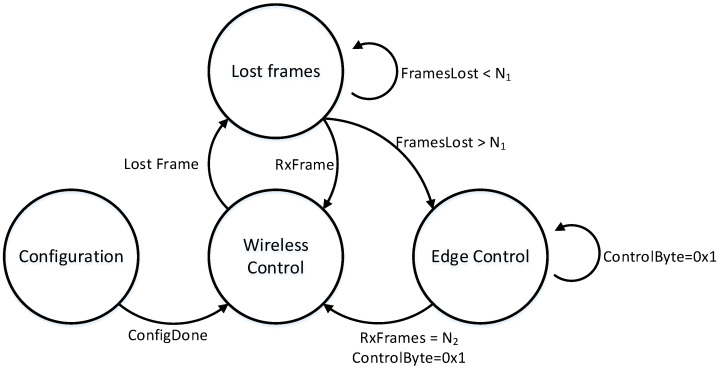
Operational states and transitions for the wireless control system.

Our approach is based on the definition of several operational states. Figure 3 shows which are these operational states and how the wireless control system may change among them. These states are:

1. **Configuration:** At start time the nodes of the system will enter in the configuration state. In this state, the Wireless controller sends the configuration to every Edge Node by means of dedicated frames. Edge Nodes receive their configuration and self-configure accordingly. The configuration includes the Edge Node identifier, the number of connected sensors and actuators and the type of connection, information to extract the control data from the wireless control frames and an offset used for arranging the sending of the measurement frames. Once, the system is properly configured and the communication links are up, the system enters into the wireless control state.
2. **Wireless control:** In this state the communication links operate appropriately. The control algorithm is executed at the Wireless Controller, and the control frames, carrying the values of the control signals, are periodically sent to the Edge Nodes. On the reception of control messages, the Edge Nodes set the value of the connected actuators and acquire the values of the sensors, which may be preprocessed at the Edge Nodes. The values read from the sensors are sent back to the Wireless Controller by means of measurement frames. In this state, Edge Nodes do not carry out any control actions, but they only act as intermediaries between the Wireless Controller and the plant.
3. **Edge control:** This state is reached when the communication links do not operate properly and the communication was assumed to be lost. In this state the control of the plant is executed at the Edge Node microcontroller by means of a low computational cost and robust control algorithm. Obviously, the performance of the system will be degraded but the process will not become uncontrolled. After N_2_ frames are received properly (see Figure 3), it goes back to the Wireless control state, assuming that communication was recovered.
4. **Lost frames:** When any frame is lost or corrupted, the system enters in this state. This state requires detecting and counting lost/corrupted frames. In this state communication problems were detected and a decision needs to be made about what to do. Thus, this is a transitory state. In this state, both the Edge Nodes and the Wireless Controller wait one interval of time (i.e., counting how many frames were lost, N_1_ in Figure 3) until it is assumed that the communication link is not operating. In case that there are just a few messages lost, the control system goes back to the Wireless Control state. Otherwise, the control system moves to the Edge control state leaving the control tasks to the Edge Nodes. While in this state, Edge Node keeps the last received value from the Wireless controller as control signal for the actuator. The control algorithm is executed at the Wireless Controller with the last value received from the Edge Nodes.

In order to simplify the deployment and execution of the switch control scheme, the same state machine was assumed for all Edge Nodes. In the future, different state machines could be used at the nodes in order to extend the flexibility of our approach. Transitions among these states are as follows: The control system is initially at the Configuration state. Once configured, when the system is able to operate, it enters in the Wireless Control state. It remains in this state while no communication problems arise. In case one frame is lost, the control system moves to the Lost Frames state. In this state, the values of the sensors and control signal will be kept unchanged, since typically the controller will be able to recover from the loss of a few frames. The system will remain in this state for a configurable interval of time, which will depend on the dynamics of the process under control. In case that the communication link is established back the system goes back to the Wireless Control state. However, if time elapses and packages are not properly received, the system moves to the Edge control state. In this state, the control is carried out by the controller programmed at the Edge node. While in this state, the Wireless Controller is sending messages to the Edge Nodes to reestablish the communication link. When the communication is operating successfully for an interval of time, the control system goes back to the Wireless Control state. Note that the values of N_1_ and N_2_ must be chosen based on the dynamics of the plant and the chosen sampling time. The lower sampling time is used and the slower the dynamics is, a higher number of communication packages may be lost without degrading the control performance. These parameters must be chosen following this rationale and checked experimentally.

### 4.2. Frames Design for Implementing the State Diagram

This section describes the frames that were used for implementing the application protocol followed in the presented wireless control approach. Namely, three types of frames are used: (1) control frames, issued by the Wireless Controller with the control information; (2) measurement frames, issued by the Edge Nodes, with the values of the process sensors; and (3) configuration frames, used at the configuration stage.

Frames were implemented using the XBee API mode, which allows higher flexibility, even at the cost of programming at a deeper level. According to the API mode, several bytes are required (e.g., *Frame delimiter*, *Length* and *Checksum*) while other bytes may be used for implementing the protocol as described below. These frames are serialized for the transmission by the XBee modules.

**Figure 4:**
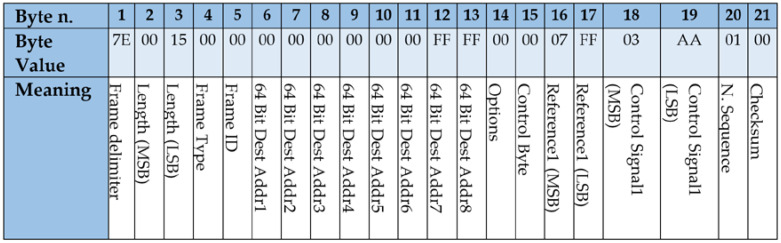
Control frame issued by the Wireless Controller to the Edge Nodes for a SISO control system.

Figure 4 shows an example of Control frame. The value of *64BitDestAddr* is used to address the frame to a specific XBee destination (e.g., 0x0013A20041AF5ADC), in case of using only one Edge Node, or to the XBee broadcast destination (0x000000000000FFFF) when several Edge Nodes are involved. Control frames are identified by the value of 0x00 in the *Control Byte*, when the system is in the Wireless Mode. If the communication is lost and the system enters in the Edge mode, the value of the Control Byte is 0x01, to indicate the Edge Node that these frames carry new values used for reconnection. For every process actuator, frames carry the values for *Reference* and *Control Signal*, using two bytes for each one. As a matter of example, Figure 4 represents a frame aimed at a SISO control system with only one actuator and one sensor. In this case, only a two-bytes value is used as *Reference*, 0x07FF, and another two-bytes value for the actual *Control Signal* sent to the actuator, 0x03AA. The value of *Reference* is sent in every frame and is used to tell the Edge Controller the value of the reference that must apply when the communication is lost. Finally, the frame includes a *N.Sequence* byte, aimed at discovering missing frames and a *Checksum* byte, aimed at detecting transmission errors. The remaining bytes must be left as they are. According to the configuration of the system, the size of the frame may vary depending on the number of actuators connected to the Edge Node.

**Figure 5:**
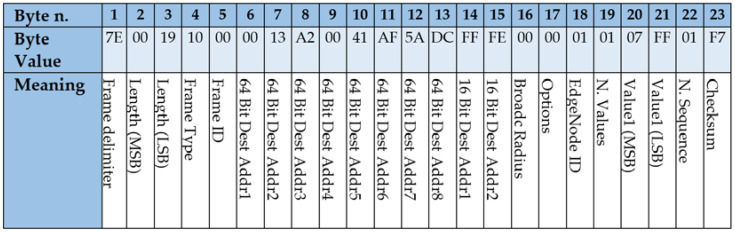
Measurement frame used by the microcontroller at the Edge Nodes with the process values for a SISO control system.

Figure 5 illustrates the structure of Measurement frames with an example for a SISO system. This frame is sent by the Edge Nodes to the Wireless Controller. This frame is addressed to the XBee address of the Wireless controller, e.g., 0x0013A20041AF5ADC. Every frame carries the Edge Node identifier, *EdgeNodeID*, which is unique, 0 × 01 in Figure 5. The frame carries the values of the sensors. In this case only one sensor value is used, *N.Values* = 0 × 01. Two bytes are used for every sensor. In the example *Value1* = 0x07FF. Finally, the frame includes *N.Sequence* for becoming aware of lost frames and the *Checksum* byte which allows identifying corrupted frames. According to the configuration of the system, the size of the frame may vary depending on the number of sensors connected to the Edge Node.

**Figure 6:**
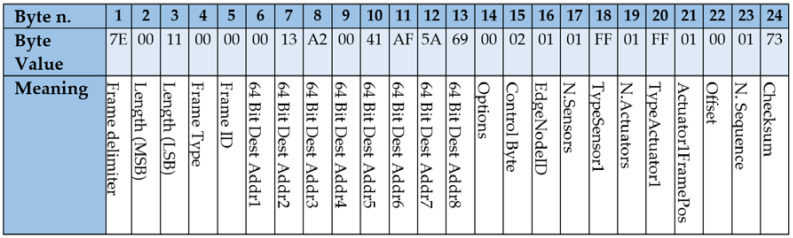
Configuration frame sent by the Wireless Controller to one Edge Node for a SISO control system.

Figure 6 shows a configuration frame issued for a SISO. In this case, frames are sent specifically to one Edge Node identified with 64BitAddr, e.g., 0x0013A20041AF5A69. Configuration frames are identified by a *ControlByte* value of 0 × 02. This frame is also used to set the logical identifier for every Edge Node by means of the *EdgeNodeID* byte, 0 × 01 in the example. Frames indicate the number of connected sensors and actuators per node by means of the *N.Sensors* and *N.Actuators* bytes. Since Figure 5 is aimed at a SISO system, only one sensor and one actuator are used, and the values are 0 × 01 in both cases. In addition, it is necessary to set the types of sensors and actuators connected to the Edge Node. This is achieved by means of the *TypeSensor* and *TypeActuator* bytes. This information must be specified for every sensor/actuator. In this example, a value of 0 × FF was chosen for both, meaning that the sensor and the actuator are connected by means of an SPI bus. Other numbers are used for specifying the pins to which the sensors or actuators are connected. The *ActuatorFramePos* is used by the Edge Node to extract the information received at the Control Frame. A value of 0 × 01 indicates that the addressed Edge Node must obtain the first actuator value issued at the control frame. It must be noted that Control frames carry the values for all actuators of the system. For that reason, in case that several actuators were used every Edge Node must be able to extract the control values for the attached actuators. The *Offset* value, 0 × 00, indicates how much time the Edge Node must wait after the reception of a Control Frame. This byte allows decoupling the transmission of the measurement frames. Finally, the frame includes a *N.Sequence* byte, aimed at discovering missing frames and a *Checksum* byte, aimed at detecting transmission errors.

### 4.3. Selection of the Control Algorithms

Our approach requires choosing adequately an Edge controller capable of being executed at the chosen Edge Node platform, which is based on an Arduino MKR Zero board. Typically, independent PIDs will be used at these nodes, since these are low computational cost controllers, but other control approaches could be used.

The Wireless Controller is implemented over a NI myRIO platform such that different sophisticated control algorithms may be implemented easily, such as Fuzzy logic, Neural Network control or MPC.

Control algorithms should be analyzed to ensure that they achieve the desired dynamics for the controlled process.

## 5. Case Study

**Figure 7:**
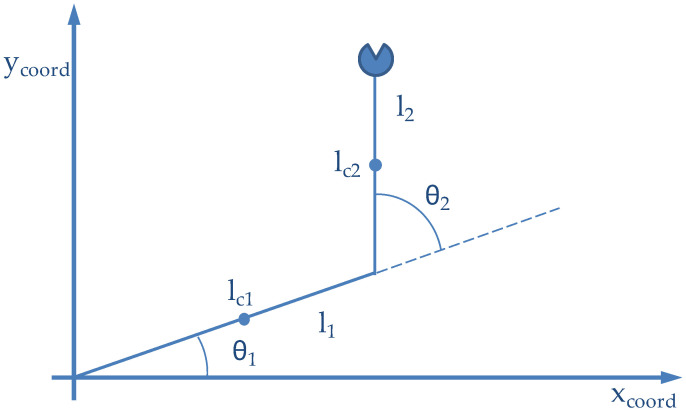
Planar manipulator with two revolute joints.

In this section, we will consider the control of the simple planar manipulator with two revolute joints as shown in Figure 7. This robot manipulator moves in the horizontal plane; thus, it is not affected by the gravitational force. The model of the system was implemented over one Arduino MKR Zero as Hardware in the Loop (HIL). The Wireless Controller, based on Fuzzy Logic, and the PID controllers implemented over the Edge Node were selected. The control scheme was validated over the experimental layout.

### 5.1. Robot Model and HIL Implementation

Let us fix the notation as follows: For each link i (i = 1,2) θ denotes the joint angle; m_i_ denotes the mass; l_i_ denotes the length; l_ci_ denotes the distance from the previous joint (i-1) to the center of mass of link i; and I_i_ denotes the moment of inertia of link i about an axis perpendicular to the plane, passing through the center mass of link i (See Figure 7)

Using the well-known Lagrangian equations in classical dynamics, one can show that the dynamic equations of this robot are:

$$\left[\begin{array}{c} \tau_{1}\\ \tau_{2} \end{array}\right]=\left[\begin{array}{cc} d_{11} & d_{12}\\ d_{21} & d_{22} \end{array}\right]\;\left[\begin{array}{c} {\ddot{\theta }}_{1}\\ {\ddot{\theta }}_{2} \end{array}\right]+\left[\begin{array}{cc} c{\dot{\theta }}_{2} & c{\dot{\theta }}_{1}+c{\dot{\theta }}_{2}\\ -c{\dot{\theta }}_{1} & 0 \end{array}\right]\left[\begin{array}{c} {\dot{\theta }}_{1}\\ {\dot{\theta }}_{2} \end{array}\right]+\left[\begin{array}{c} f_{1}\\ f_{2} \end{array}\right]$$

where the coefficients $d_{ij},\;f_{i}$ and c are as follows (note that the $b_{i}$ are the viscous friction coefficients):

$$d_{11}=m_{1}l_{c1}^{2}+I_{1}+m_{2}\left[l_{1}^{2}+l_{c2}^{2}+2l_{1}l_{c2}\cos\theta_{2}\right]+I_{2}\;d_{22}=m_{2}l_{c2}^{2}+I_{2}\;d_{12}=d_{21}=m_{2}l_{1}l_{c2}\cos\theta_{2}+m_{2}l_{c2}^{2}+I_{2}\;c=-m_{2}l_{1}l_{c2}\sin\theta_{2}\;f_{1}=b_{1}{\dot{\theta }}_{1}\;f_{2}=b_{2}{\dot{\theta }}_{2}$$

The values for the robot parameters shown in Table 6 were assumed in the HIL implementation:

This model was implemented as HIL plant over an Arduino MKR Zero. The connection between the Edge Node and the HIL plant was implemented by means of the SPI bus. Namely, two variables were used to set the control values used as actuators and the current values for the position of the joints of the robot. This layout emulates the behavior of the Wireless Control Scheme when a real plant is used and was chosen for validating the presented approach.

### 5.2. Design of the Local and Remote Controllers

For validation purpose, two different control algorithms were chosen to control the planar manipulator described above and implemented as HIL plant. Although PID controllers provide good performance for a broad number of plants, in non-lineal systems, such as the presented robot, they should be tuned at different points of operation. In this work two control approaches were used. A more sophisticated controller, with higher computing requirements, was executed at the Wireless Controller, whereas two PID controllers were executed at the Edge Nodes as backup controllers in case of communication failures. These PID controllers were tuned to operate in a wide range. A sampling time of 30 ms was chosen since this may be a viable sampling time according to the experimental validation of the XBee 900 MHz modules while being compatible with the dynamics of the planar manipulator.

PID controllers are commonly used as feedback controllers in several industrial applications due to their simplicity for implementation; additionally, these are suitable for being used over low resource computing platforms [56]. It is estimated that one-third of industrial plant control loops use PIDs due to its reliability [57]. For this reason, it was selected to be implemented as backup controllers in the Edge Nodes when the communication link is lost. Below it is represented the common expression for this control scheme, where *e* is the measured error at each step *k* with a sampling time Δ*t* (30 ms); the variables *K_p_*, *K_i_* and *K_d_* are, respectively, the gains of the proportional, integral and derivative terms [58].

$$u\left(k\right)=K_{p}e\left(k\right)+K_{i}\overset{k}{\underset{i=1}{\sum }}e\left(i\right)\Delta t+K_{d}\frac{e\left(k\right)-e\left(k-1\right)}{\Delta t}\;$$

Namely, two PID controllers, one per each robot joint, were tuned. Both PIDs controlled the movement of every robot joint independently. The controller gains are displayed in the Table 7.

**Figure 8:**
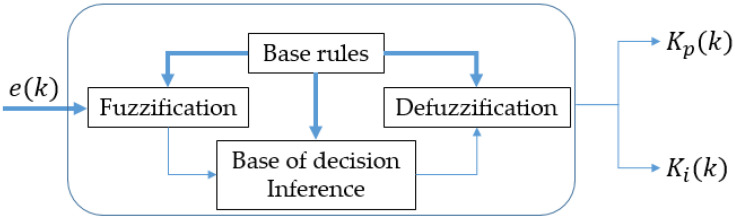
Fuzzy structure for PI gain scheduling.

Major downsides of PIDs are related to the strategies to obtain the appropriate gains to prevent oscillations, which can yield to the instability in complicated non-linear or time-varying systems [59]. A sophisticated approach that can improve an ordinary PID is through a gain scheduling strategy based on a Fuzzy Logic Control (FLC) structure [60]. Despite that this framework requires higher computing capabilities it may achieve better control results in some systems, especially in non-lineal systems. For these reasons, a Fuzzy controller was designed to be implemented at the Wireless Controller as a gain scheduler based on Fuzzy Logic for a PID Controller. FLC is a knowledge-based method that is configured through an operator expertise on a particular system [61]. The input information is fed into a fuzzification block that translates the numerical values to linguistic terms (also called *membership functions*) defined by the designer; subsequently, these are evaluated in IF–THEN rules. As a consequence, the result is employed at the inference for the activation in the defuzzication process, which outputs gains for the PID controller [62]. In this work, a gain scheduling of the proportional and integral terms was arranged in the FL set up as Figure 8 shows. The derivative gains were left at constant values of 6.5 and 0.6, respectively. Table 7 shows the range gains obtained with this approach.

**Figure 9:**
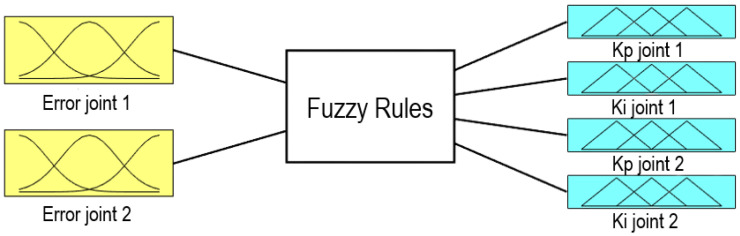
Fuzzy controller configuration within inputs and outputs.

The input membership functions for the error of every joint of the robot were set in a triangular overlapped arrangement. Additionally, these triangular functions were uniformly discretized as negative big (NB), negative medium (NM), negative small (NS), zero (Z), positive small (PS), positive medium (PM) and positive big (PB). Likewise, the output membership function was configured with the same geometrical conditions. Through several iterations in simulations to achieve expertise in the configuration of the gains, the geometrical settle was used as low values when the error is high and vice versa. A schematic description of the FLC arrangement is displayed in Figure 9.

**Figure 10:**
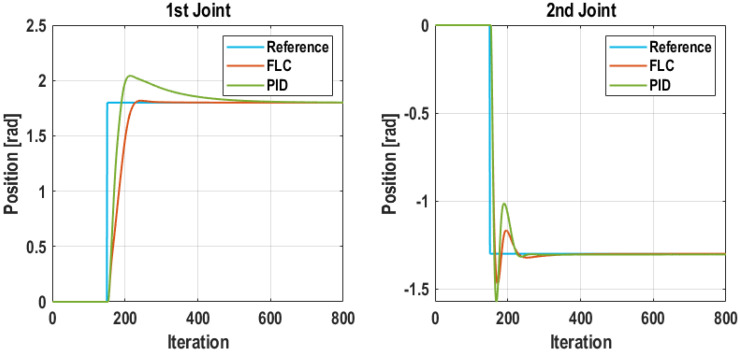
Response comparison to step reference of PID and Fuzzy Logic Controller over the robot HIL model.

In order to compare the performance of both controllers, they were implemented over the robot HIL model of the plant. A step response was chosen to evaluate the performance of PID and FLC control schemes, since step references are typically used as benchmark to compare the performance of different control schemes. This approach provides a critical and dynamical analysis for the proposed frameworks; outcomes are exhibited in Figure 10. Despite that the PID has a faster response, the FLC counteracts with a negligible overshoot and lower settling time. At the same time, in the second joint, the PID carries with a higher overshoot, whereas the FLC behaved better in this sense as the amplitude is reduced to almost the half. In addition, the integral of the absolute error (IAE) was calculated to inspect the advantages in numerical terms that are shown in Table 8 where the FLC carries with the assets. Based on this analysis, it is found that the proposed FLC is a suitable advanced controller for further tests. This is the control scheme implemented at the Wireless Controller. On the other side, the tuned PID controllers were implemented as Edge Controllers to be used as backup controllers in case that the communication link is lost.

### 5.3. Experimental Tests

Several tests were used for validating the presented wireless control approach under different situations. The model for the planar manipulator with two revolute joints was executed as HIL in one Arduino MKR Zero. For all tests, a sampling time of 30 ms was chosen. Tests lasted 27 s, using a total of 900 samples. This was considered a reasonable duration for this robot to reach the permanent state and checking that it remained in this position. The distance between the Wireless Controller and the Edge Node was of one meter.

In order to evaluate the impact of lost messages in the performance of the wireless control scheme communication faults were injected at selected time instants. Fault injection consisted of defining of purpose when frames were not sent by the Wireless Controller. Thus, it is possible to evaluate the behavior of the wireless control system in case of loss of frames. Two types of fault injections were used: Sporadic loss of one frame and bursts of lost frames. A number of five lost frames were used as N_1_ value in the state diagram depicted in Figure 3 to trigger the state change from the *Lost frames* to the *Edge Control* states. The same number, N_2_, was used to trigger the change from the *Edge Control* state back to the *Wireless Control* state.

The FLC is always executed at the Wireless Controller, and the PID is always executed at the Edge Controller. In most tests the reference trajectory was generated at the Wireless Controller and the controller evaluated the control signal accordingly. However, when the control system went into the *Edge Control* state the reference was changed since it was not possible to send a smoother trajectory for the PID algorithm.

The value of the Integral Absolute Error (IAE) for each joint was calculated to compare the performance of the wireless control scheme under different circumstances.

#### 5.3.1. Cubic Trajectory without Injected Communication Errors

**Figure 11:**
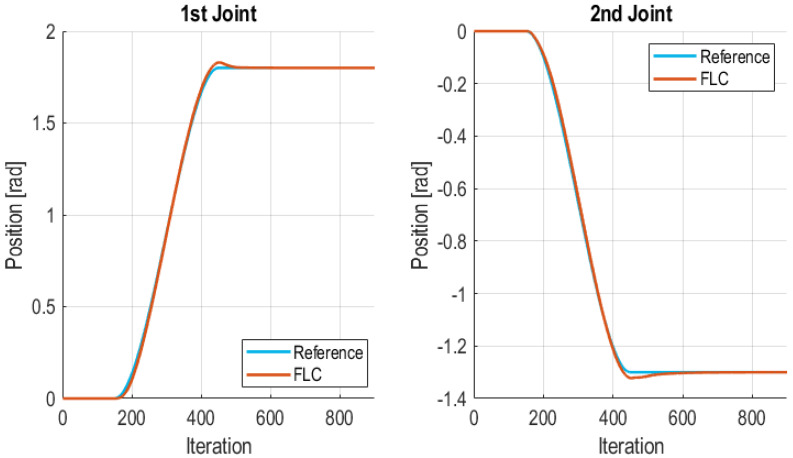
FLC for a cubic trajectory without communication errors.

Cubic polynomial trajectories are typically used for moving robotic arms since they provide continuity in both position and velocity, producing smooth movements which are less aggressive for the robot mechanics [63]. These trajectories are typically used for controlling the movement of robotic arms [64]. However, step references are used at the Edge Nodes when the communication between the Wireless Controller and the Edge Nodes is degraded or lost. Step trajectories allow that Edge Nodes control locally the plant to reach the final position of the joint at a low computational cost even using occasionally a more aggressive trajectory. Thus, the cubic trajectory is only generated at the Wireless Controller. This section evaluates the performance of the FLC, executed at the Wireless Controller, when a cubic trajectory planning is chosen. In this test communication errors were not injected; thus, it can be taken as reference test. The values of the IAE for each joint of the manipulator are 5.7275 for the 1st joint and 5.4578 for the 2nd joint. Figure 11 shows the result of the experiment for both joints. It can be appreciated that the FLC is able to follow the cubic trajectory for both joints of the robot without permanent error and with a good transitory response.

#### 5.3.2. Cubic Trajectory with Sporadic Communication Errors

**Figure 12:**
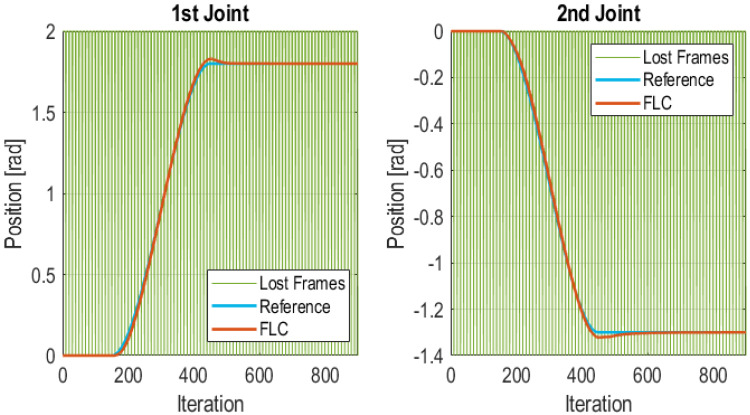
FLC for a cubic trajectory with sporadic communication errors.

In this test control frames are periodically lost. But only one frame is lost. Thus, the controller enters in the *Lost frames* state. While in this state, the Edge Node uses the last received value from the Wireless Controller as control signal for the actuator. The control algorithm is executed at the Wireless Controller with the last value received from the Edge Nodes. In this test, one frame was not sent every 10 sample times. Thus, the percentage of lost packets was around 10%. Figure 12 shows the behavior of the system in this situation, and the instants when packets were lost, as vertical green lines. It can be appreciated that with such a high percentage of lost packets, the performance of the Wireless Control Schema was satisfactory. The values of the IAE for each joint of the manipulator are 5.7453 for the 1st joint and 5.4581 for the 2nd joint.

#### 5.3.3. Burst Frames Loss at Stationary State

**Figure 13:**
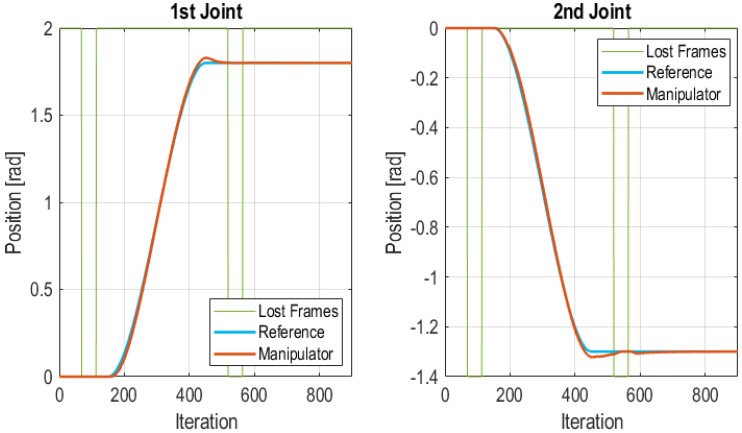
FLC for a cubic trajectory with burst of lost frames while in steady state.

In this test a burst of frames was lost when the system had reached a stationary position. Bursts of 45 lost frames were injected, i.e., 1.35 s without receiving any frame. As a consequence, in this test, the system changed among the states of the state diagram depicted in Figure 3. It went through the following state sequence: (1) Wireless Control; (2) Lost frames; (3) Edge control and (4) Wireless Control. The total number of lost frames in the test was of 90 frames which accounts for the 10% of total frames. The use of the PID, executed at the Edge Controller while in the Edge Control state, operated with a reference value that let the system remain stationary. Obtained results were satisfactory. The values of the IAE for each joint of the manipulator were 5.7275 for the 1st joint and 5.4507 for the 2nd joint. These values were similar to the values obtained without injected communication errors. Figure 13 shows the two regions where frames were lost, the first region between iterations 65 and 110 and the second between iterations 520 and 565. The limits of both lost frames intervals are shown in Figure 13 with green lines.

#### 5.3.4. Burst Frames Loss at Beginning of Transitory State

**Figure 14:**
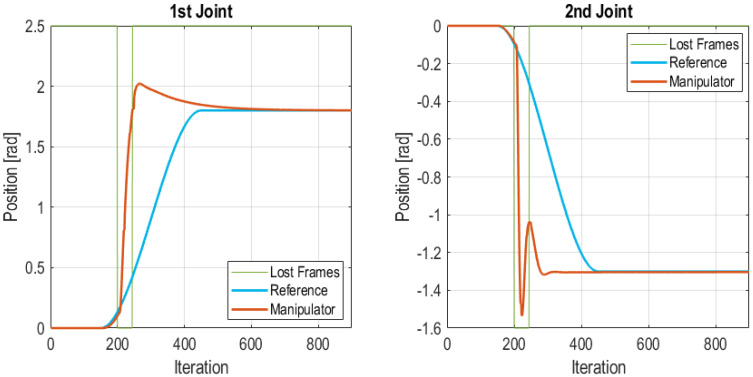
Burst of frames lost at start of the transitory state.

In this test a burst of frames is lost just at the moment when the system is requested to move both joints. Namely, a burst of 45 lost frames was injected between iteration 200 and 245, totaling 1.35 s. The interval in which frames were lost is bounded with green lines in Figure 14. In this case, when the Edge Node detects that the communication is lost the Edge Controller takes over. The PID algorithm uses the reference value set in the control frame (see Figure 4) as final value for a step reference. The consequence of this fact is that the reference is changed. However, as Figure 14 shows the Edge Controller is able to move the robot to the desired position, although it follows a more aggressive trajectory for reaching the final position. In this case, the Edge Node goes through the following state sequence: (1) Wireless Control; (2) Lost frames; (3) Edge control. The Edge Controller remains in operation until it reaches a stationary position. At this instant it may enter back to the Wireless Control state. The total number of lost frames in the test was of 45 frames which accounts for the 5% of total frames. The IAE values for each joint of the manipulator were 174.50 for the 1st joint and 115.52 for the 2nd joint. It is important to note that this is the worst case performance for the proposed wireless control scheme, since the desired trajectory, a cubic trajectory, is compared with the default trajectory, a step trajectory with the same final value. When tuning the PID it was checked that this dynamics is acceptable for the manipulator.

#### 5.3.5. Burst Frames Loss at the Middle of Transitory State

**Figure 15:**
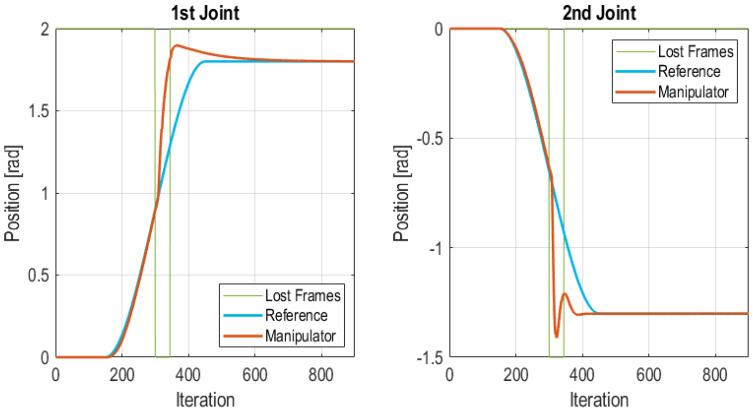
Burst of frames lost at the middle of transitory state.

In this test, a burst of frames is lost in the middle of a transition from one position to another. A burst of 45 lost frames was injected between iteration 300 and 345, i.e., a total of 1.35 s. The interval in which frames were lost is bounded with green lines in Figure 15. In this case, when the Edge Node detects that the communication is lost and the Edge Controller takes over, the PID uses a step reference in which the final value is extracted from the reference value set in the control frame (see Figure 4). However, as Figure 15 shows the Edge Controller is able to move the robot to the desired position, although it follows a different trajectory for reaching the final position, since the reference is changed at the moment that communication is lost from a cubic trajectory to a step trajectory. In this case, the Edge Node goes through the following state sequence: (1) Wireless Control; (2) Lost frames; (3) Edge control and remains in this state until it reaches a stationary position. In this point it enters back to the Wireless Control state. The total number of lost frames in the test was of 45 frames which accounts for the 5% of total frames. The IAE values for each joint of the manipulator were 48.94 for the 1st joint and 31.36 for the 2nd joint. These values are considerably lower than the values obtained in test Section 5.3.4.

#### 5.3.6. Burst Frames Loss at the End of Transitory State

**Figure 16:**
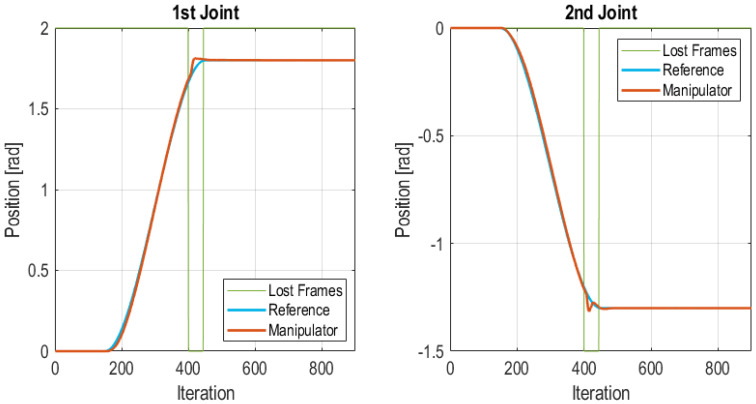
Burst of frames lost at the end of transitory state.

In this test, a burst of frames is lost at the end of a transition from one position to another. A burst of 45 lost frames was injected between iteration 400 and 445, i.e., a total of 1.35 s without communication. The interval in which frames were lost is bounded with green lines in Figure 16. In this case, when the Edge Node detects that the communication is lost and the Edge Controller takes over, the PID uses as step reference the value extracted from the reference value set in the control frame (see Figure 4). The consequence of this fact is that the reference is changed at the moment that communication is lost. However, as Figure 16 shows the Edge Controller is able to move the robot to the desired position, although it follows a different trajectory for reaching the final position. Again, the Edge Node goes through the following state sequence: (1) Wireless Control; (2) Lost frames; (3) Edge control and remains in this state until it reaches a stationary position. However, in this case the effect of the reference and controller change at the system is small, since the time that both references are different is short. The total number of lost frames in the test was of 45 frames which accounts for the 5% of total frames sent. The IAE values for each joint of the manipulator were 5.42 for the 1st joint and 3.89 for the 2nd joint. 

#### 5.3.7. Communication Recovery when Step Reference Is Used

**Figure 17:**
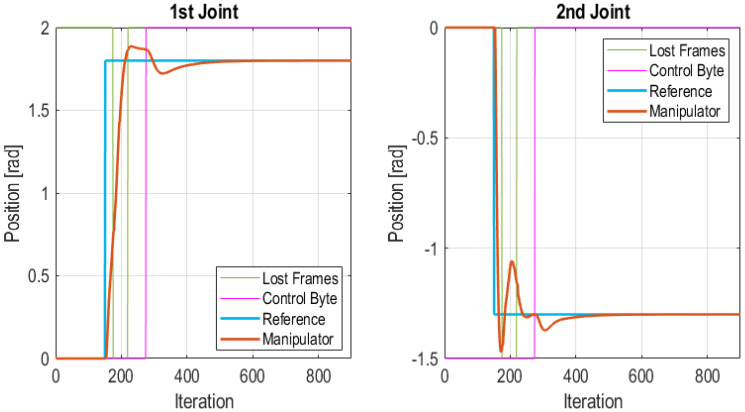
Communication recovery when a step reference is used.

In previous tests, the communication between with the Wireless Controller was restored when the system reached a steady state. This was performed because there was a change in the reference signal. This test evaluates the performance of the controller when both the FLC and the PID use the step reference and the communication is restored. In this test a burst of lost frames is produced between iterations 175 and 210 at the beginning of the transitory state. The interval in which frames were lost is bounded with green lines in Figure 17. However, in this case, the communication is restored at iteration 275. The limits of the lost frames interval are shown in Figure 17 with green lines. Figure 17 shows the performance of the Wireless Control approach for this test. Results prove that the performance of the approach is satisfactory in this case. The IAE values for each joint of the manipulator were 65.96 for the 1st joint and 25.37 for the 2nd joint. These are similar values to the IAE values obtained when a step reference is used with either the PID or the FLC controllers, see Table 8.

#### 5.3.8. Discussion

Table 9 summarizes the IAE values for all carried tests aimed at validating the wireless control scheme. A step reference was taken as benchmark to compare the performance of both the PID controller, implemented at the Edge Nodes as backup controller, and the FLC implemented at the Wireless Controller as main controller. The IAE shows that the performance of the second controller is better, but it needs higher computational cost. A cubic trajectory was used, since it is less aggressive for the robot mechanics, but the step trajectory was also considered as acceptable. The control scheme behaves adequately for the cubic trajectory, and the results proved little influence when sporadic message dropouts occurred. In addition, the performance of the wireless control scheme was adequate when burst frames loss occurred. Results proved that the plant did not become uncontrolled and that it reached the desired reference position. This was especially true when the system was in a stationary state. The value of the IAE was similar to previous values for the cubic trajectory. In case the system is in transitory state, the values of the IAE prove that the worst situation is when the burst loss occurs just at the beginning of the transitory state. This is in part due to the change in the reference trajectory that the PID executes. When the burst loss occurs in the middle the IAE values are better and when it is at the end the IAE results are comparable to those obtained for the cubic trajectory. These results validate the approach for being used at the chosen robot manipulator.

## 6. Conclusions

Wireless technologies are being accepted in production processes since they introduce diverse benefits, such as introducing deployment flexibility, supporting mobile devices or reducing cabling. Although these technologies are starting to be introduced in some industrial operations, wireless links are less predictable that wired links. The nature of wave propagation, which introduces some phenomena, such as signal fading or interferences, reduces the QoS parameters of the communication links. These facts may reduce the application of wireless technologies to close-loop applications. In addition, wireless control systems must be designed in a holistic way since both the control algorithms and the communications performance are intertwined. Some communication parameters such as latency, jitter of bit error rate influence the available sampling period and must be considered in the design of the control algorithm. In addition, the control algorithms must be able to cope with QoS degradation or even a total loss of the wireless communication link. 

In this scenario, this work presents a wireless control scheme aimed at achieving reliable wireless control systems even in the case of communication degradation or total loss of the communication link. The presented control approach is based on the use of two levels of controllers: sophisticated control algorithms, providing better performance, are executed in a central node, while back controllers, implemented as local independent controllers, are executed next to the process in case of communication degradation. An operating architecture with two types of nodes was defined, the so-called Wireless Controller and Edge Nodes. The Wireless Controller, implemented in a central node, is able to execute sophisticated algorithms, requiring higher computing resources. Edge Nodes, located next to the process, will take over in case of QoS degradation by executing back controllers implemented over low computing resources platforms. The presented approach presents a reliable strategy for switching between central and local controllers avoiding that the system becomes uncontrolled and recovering the central controller when the communication QoS parameters are considered safe. For validation purposes, the control scheme was implemented over a NI myRIO 1900 as Wireless Controller and an ad hoc board powered with an Arduino MKR Zero as Edge Node. The design of the application protocol used by these devices is also discussed.

An independent communication channel was used for control communication operations. Namely, XBee 900 MHz modules were used. This technology minimizes the interferences caused by other technologies, typically operating at the 2.4 GHz band [12,13], and leaving the 2.4 GHz band available for connecting to cloud services with less-critical requirements. In this work, XBee 900 MHz modules were validated for control applications by means of several experimental tests. In addition, an application protocol was designed on top of this technology to support the proposed wireless control scheme and the switching between the Wireless Controller and Edge Nodes Controllers.

The presented approach was validated by means of a planar robot, emulated as Hardware in the Loop plant and connected to the Edge Node by means of the SPI bus. An advanced control algorithm, based on Fuzzy Logic, was executed at the Wireless Controller, whereas a low resource PID was executed as back controllers at the Edge Nodes. Experimental results proved that the planar robot did not become uncontrolled, even in case of QoS degradation or total communication loss. These results may be extended to other plants, such that the current case study may serve as a guide to introduce wireless technologies to critical control applications.

Several issues will be developed in future works. Namely, further experiments related to realistic scenarios are proper to consider for future research. Since XBee 900 MHz modules may operate at longer distances, its applicability to control applications at longer distances must be analyzed. Another issue to analyze in the future is the stability and performance of the system depending on the number of frames lost. Finally, the applicability of some of the concepts proposed in this work can be evaluated over different technologies such as WirelessHART.

## Figures and Tables

**Table 1:** Configuration parameters of the XBee modules.

XBee Configuration Parameter	Value
CE (Routing/Messaging Mode)-*Wireless Controller*	Indirect Message Coordinator
CE (Routing/Messaging Mode)-*Edge Nodes*	Indirect Message Poller
NH Network Hops	1
MR Mesh Unicast Retries	0
RR (Unicast Retries)	0
NN Network Delay Slots	1
RR (Unicast Retries)	0
PL (TX Power Level)	Highest (+24 dB)
Baud Rate	115,200

**Table 2:** Maximum and minimum latency values and jitter for the XBee messages issued by the Wireless Controller.

Distance (m)	Minimum (ms)	Maximum (ms)	Jitter (ms)
0	9.62	10.60	0.98
1	9.68	10.62	0.94
5	9.64	10.64	1
8	9.68	10.62	0.94
12	9.66	11.42	1.76

**Table 3:** Lost/wrong frames at different distances even with a brick wall in between.

Distance (m)	Lost/Wrong Frames	Percentage
0	4	0.02%
1	2	0.01%
5	2	0.01%
8	3	0.015%
12	13	0.065%
1 (brick wall)	0	0%
5 (brick wall)	4	0.02%
8 (brick wall)	5	0.025%
12 (brick wall)	24	0.12%

**Table 4:** Reception jitter at three different Smart Measuring Nodes at different distances.

Distance (m)	Jitter (ms)
0	0.76
1	0.76
5	0.72
8	0.84
12	0.96

**Table 5:** Reception timestamps of Smart Measuring Nodes at the Coordinator Node (in ms).

Frame Size	Minimum (ms)	Maximum (ms)	Jitter (ms)
23 bytes	11.44	11.94	0.5
29 bytes	12.68	13.28	0.6
37 bytes	14.5	15.34	0.84
53 bytes	17.7	18.7	1

**Table 6:** Robot parameters implemented in the HIL.

	1st Joint	2nd Joint
m_i_ (Kg)	2.0	1.0
l_i_ (m)	1.0	0.7
l_ci_ (m)	0.4	0.3
I_i_ (Kg·m^2^)	0.25	0.1
b_i_ (Kg/s)	1.0	1.0

**Table 7:** Gain table for the conventional PID and the Gain Scheduling PID tuned.

		*K_p_*	*K_i_*	*K_d_*
Conventional PID	1st joint	20	5	10
	2nd joint	10	7	2
Gain Scheduling PID	1st joint	0–30	0–18	6.5
	2nd joint	0–12	0–9	0.6

**Table 8:** IAE for both controllers: PID at the Edge Node and FLC at the Wireless Controller.

	PID	FLC
Joint 1	69.37	57.26
Joint 2	23.46	18.11

**Table 9:** Summary of IAE for all tests.

Test	Controller	1st Joint	2nd Joint
Step Reference—no dropout	PID	69.37	23.46
Step Reference—no dropout	FLC	57.26	18.11
Cubic trajectory—no dropout	FLC	5.73	5.46
Cubic trajectory—sporadic dropouts	FLC	5.74	5.46
Burst frames loss—stationary state	FLC-PID	5.73	5.45
Burst frames loss—beginning of transitory state	FLC-PID	174.50	115.52
Burst frames loss—middle of transitory state	FLC-PID	48.94	31.36
Burst frames loss—end of transitory state	FLC-PID	5.41	3.89
Step reference—communication recovery	FLC-PID-FLC	65.96	25.38

## Data Availability

Not applicable.
